# Does Accountability Aggravate the Risk of Teacher Burnout? Evidence from the Chinese Education System

**DOI:** 10.3390/bs13090772

**Published:** 2023-09-15

**Authors:** Guanyu Li, Kwok Kuen Tsang

**Affiliations:** 1 College of Educational Administration, Beijing Normal University, Beijing 100875, China; gli64@outlook.com; 2 Department of Education Policy and Leadership, The Education University of Hong Kong, Hong Kong 999077, China

**Keywords:** external accountability, internal accountability, emotional labor, burnout, teacher

## Abstract

External and internal accountabilities and emotional labor are possible factors triggering teacher burnout, but the relationships between the constructs have not been fully addressed. Thus, this study aimed to construct and test a chain mediation model to demonstrate the relationships between external accountability and burnout, mediated by internal accountability and emotional labor sequentially. By surveying 814 teachers (years of teaching: M = 13.42, SD = 10.97) from 10 provinces in China, it shows: (1) external and internal accountabilities are both negatively related to burnout; (2) internal accountability, deep acting, and expression of naturally felt emotions mediate the relationship between external accountability and burnout; (3) internal accountability and surface acting sequentially play a chain mediating role between external accountability and burnout; and (4) internal accountability and expression of naturally felt emotions sequentially play a chain mediating role between external accountability and teacher burnout.

## 1. Introduction

As influenced by neoliberalism, the idea of minimizing state intervention through decentralization of public sectors has spread worldwide since the 1980s. In this neoliberal context, one of the notable characteristics of public education systems is the emphasis on holding teachers accountable to stakeholders such as students, students’ parents, and school and state authorities [1]. In this sense, although educational decentralization is valued, education systems tend to restrict rather than enhance teachers’ power, thereby making them vulnerable to external control and work, resulting in teachers’ stress, emotional exhaustion, and depersonalization [2]. Therefore, prior studies suggest accountability as a significant factor contributing to teacher burnout [3]. Nevertheless, these studies have a limitation in that they only refer to accountability as external accountability, as a managerial mechanism based on measures such as performance indicators and standardized assessment, holding teachers answerable for their decisions, actions, and outcomes. However, they take insufficient account of internal accountability as a normative mechanism based on a virtue in accordance with expectations and demands of professional values and ethics that encourage teachers’ self-governance with little external security [4]. It is noted that internal accountability is an equally important mechanism, disciplining the behaviors and mentalities of teachers alongside external accountability [5]. In many societies, such as the United States, Australia, and Hong Kong, governments initiate measures such as teachers’ professional and competency standards to promote the internal accountability of teachers as a key element of education reforms in the neoliberal era [6]. In other words, teachers should be subject to both external and internal accountabilities so that studies that are concerned only with external accountability alone do not provide a complete analysis of the impacts of accountability on teacher burnout. Moreover, another limitation of the prior studies is that they provide insufficient examinations regarding the roles of emotional labor in the relationships between accountability and teacher burnout. As emotion management is performed for wages or work goals, emotional labor should affect teacher burnout and be affected by external and internal accountabilities [7]. In other words, emotional labor should mediate the effects of both forms of accountability on teacher burnout, but the mediation effects of emotional labor have not been sufficiently examined in the literature. Accordingly, the present study extends these existing studies, but is original and significant, through the investigation of the relationship between external and internal accountabilities, emotional labor, and teacher burnout, thereby filling the research gaps.

The present study focuses on the context of China. Similar to its international counterparts, the public sector reform of China has been influenced by neoliberalism [8]. As a central characteristic of the political system, accountability is regarded as the technology of governance to recentralize the delegated power through neoliberal education policies [9]. Thus, there are extensive external accountability measures for teachers’ work, such as educational inspections, teacher performance evaluation, and performance-pay, to monitor teachers’ work [10]. Moreover, the central government has reformed the teacher appraisal system to effectively evaluate teachers’ professional values and ethics (*shide*) through several policy documents, such as *the Opinion on Deepening Reform of Teacher Professional in the New Era* in 2018, and *the Opinion on Strengthening the Development of Teacher Professional Ethics in the New Era*, published by the Ministry of Education in 2019. In this sense, the government has attempted to managerially promote and define the professional values and ethics of teachers, according to the above, to ensure teachers’ behaviors are in accordance with the state’s expectations and requirements, leading to feelings of meaninglessness and powerlessness among teachers [9]. Accordingly, teachers in China have been extensively held externally and internally accountable by the state. In this situation, it is estimated that 1/4 of teachers are burned out in China [11]. As a result, China is a suitable context for examining the relationship between accountability and teacher burnout. The findings may have implications for international communities seeking to alleviate teacher burnout by improving accountability systems for teachers.

## 2. Burnout

The concept of burnout was coined by Freudenberger [12] to describe feelings of being “worn out” due to job conditions such as high job pressures and demands and routinization of the job. Based on Freudenberger’s work, Maslach et al. [13] further developed the concept and suggested a three-dimensional model of burnout. According to the model, burnout consists of emotional exhaustion, as feelings of being emotionally overextended and drained; depersonalization, as feelings of being cynical and detached from one’s work or other persons at work; and reduced personal accomplishment, as a decreased sense of competence, efficacy, and achievement. According to the literature, burnout is affected by a variety of risk factors, such as work overload, that make it hard for people to rest, recover, and restore balance. This lack of control restricts people’s autonomy and power to do their work in line with their preferred procedures and personal values, and insufficient rewards that devalue people’s efforts at work [14].

## 3. External Accountability and Teacher Burnout

Labor process theory is frequently cited for the analysis of how teachers become alienated from their labor process, causing them to be at risk of burnout in the neoliberal era [15]. According to the theory, teachers are at risk of burnout because education systems introduce the logic of external accountability into education management to prescribe and standardize the procedures, methods, and outcomes of teachers’ work, and make teachers answerable and responsible for their actions and decisions to stakeholders [16]. In this sense, teachers are deskilled to determine and decide the labor process of teaching but subject to external controls exercised by stakeholders [17]. Deskilling implies vulnerability to extra duties outside the classroom assigned by state and school authorities, leading to work intensification [18]. The literature has suggested that teachers currently have to handle numerous administrative and noninstructional duties, such as paperwork and documentation, report writing, and promotion and management of the school’s public image, in addition to instructional work due to external accountability [19]. Thus, teachers become prone to stress and exhaustion because of excessive workload.

In addition, external accountability can demoralize teachers [20]. The literature indicates that moral purpose, such as making a difference for students, is usually the guiding force for people, including the Chinese, to choose to teach [21]. Therefore, when they find that they can reach their moral purpose through their work, they feel intrinsically rewarded, leading to positive emotions [22]. Nevertheless, when deskilled, they lack control over their work and are only able to do their work in the prescribed ways in terms of standardized frameworks of external accountability. The prescribed ways may not fit their moral purposes and professional values, leading to a sense of depersonalization and reduced personal accomplishment [14]. For instance, Anagnostopoulos [23] found that high school teachers in Chicago perceived that district policies, which held them accountable for students’ test scores, forced them to prioritize teaching for the tests over taking care of students’ all-round development. In this situation, teachers may find their work alienating, as their moral purposes and efforts are not valued and appreciated by others and the education system [2]. As a result, they may treat their work as just a job to earn a living, with less intrinsic rewards.

Accordingly, external accountability may put teachers at risk of burnout because it tends to deskill and disempower teachers, leading teachers to have a lack of control over their work and intensifying their work, resulting in excessive workload and stressful work conditions, and demoralizing them, leading to insufficient intrinsic and psychic rewards. Therefore, the following hypothesis is formulated:

**Hypothesis 1 (H1).**  *External accountability is positively related to teacher burnout*.

## 4. Mediating Effect of Internal Accountability

Although external accountability is underlined in the neoliberal context of education, teachers still enjoy a certain degree of power to implement the work decided by the state and school authorities within a standardized framework due to the initiatives of educational decentralization such as school-based management [24]. Nevertheless, labor process theory argues that decentralization initiatives are the technology that disciplines teachers to govern themselves based on predefined social norms and expectations [25]. In other words, when teachers are delegated a greater degree of power by the education system, they have a greater sense of responsibility to do the work and achieve the work goals according to a standardized framework of teachers’ work [26]. In this condition, teachers become self-governing bodies who reflexively perform the work within the standardized framework. Nevertheless, labor process theorists (e.g., [27]) note that self-governing capability can lead to resistance, as it allows individuals to develop their own subjectivity, which may challenge the dominant authorities who are constructing stressful and alienated work conditions. Therefore, to avoid resistance, state authorities exercise a normative mechanism to discipline teachers’ subjectivity to ensure consent with the standardized framework that they prescribe [16]. The normative mechanism can be conceptualized as internal accountability, and thereby a virtue related to professional values and ethics serving as inner standards for self-regulation with little external security [4].

According to Rosenblatt [4], professionalism is the essential disposition of internal accountability disciplining teachers’ subjectivity. Teacher professionalism generally concerns values and ethics, such as caring for students and committing to the school and work [28]. Professionalism is generally internalized into teachers’ professional self, holding them self-accountable for their decisions and actions. Therefore, they may take internal accountability for granted and even perceive it as a source of self-autonomy for meaningful work [29]. In other words, as Qin [30] found, when internal accountability is high, teachers have a stronger sense of self-autonomy. Thus, internal accountability may help alleviate teacher burnout.

Due to its negative association with burnout, from the perspective of labor process theory, internal accountability may help reduce teachers’ critical awareness of the deskilling and disempowering effects of external accountability and, ultimately, legitimize the standardized framework for them, leading to greater tolerance of stressful and alienating work conditions [26]. The disciplinary function of internal accountability becomes more obvious when the state authorities promote new meanings of teacher professionalism based on neoliberal values of education, such as standardized and measurable outcomes [31]. In the new concept of professionalism, teachers are deemed responsible for producing desirable educational outcomes that are evidenced by objective indicators such as exam scores [9]. If they failed to achieve this, they would be perceived as unprofessional [6]. As a result, internal accountability may be accounted for externally.

Accordingly, internal accountability may be negatively associated with teacher burnout but positively related to external accountability. In this sense, it may play a mediating role between external accountability and teacher burnout. Therefore, the following hypothesis is formulated:

**Hypothesis 2 (H2).**  *The relationship between external accountability and teacher burnout is mediated by internal accountability*.

## 5. Mediating Effect of Emotional Labor

The literature has suggested that emotional labor is an essential part of teachers’ work because teachers need to manage their own emotions and the emotions of students, and even the emotions of students’ parents, to create and maintain positive environments for effective teaching and learning [32]. To achieve their work goals, they can employ emotional labor strategies, including surface acting (the effort of displaying required emotions by hiding felt emotions or faking unfelt emotions), deep acting (the effort of displaying required emotions by modifying felt emotions with cognitive techniques such as distraction and self-persuasion), and the expression of naturally felt emotions (the effort of spontaneously displaying genuine emotions) [33].

Surface acting and deep acting are positively associated with teacher burnout [34]. Hochschild [35] implies that emotional labor, especially surface acting and deep acting, depletes employees’ emotion. As people are generally employed by bureaucracy—teachers are employed by state or school bureaucracy—in postindustrial societies, their emotions tend to be bureaucratically regulated to satisfy and fulfill bureaucratic interests and goals rather than to express their values. Thus, to earn a living, they have to consciously manage their feelings and displays according to bureaucratic regulations [34]. In this situation, they are at risk of emotive dissonance due to the separation of authentic feelings and displayed feelings [36]. On the other hand, the expression of naturally felt emotions should be positively associated with teacher burnout because it is autonomous and spontaneous emotion management that requires less effort to modify feelings or displays, resulting in emotive consonance [33] and a sense of authenticity [37].

The pattern of teachers’ emotional labor depends on the degree of power teachers enjoy in teaching [36]. If they have a greater degree of power, they are more capable of expressing their genuine emotions due to having less stress and fewer demands on emotion management from bureaucratic regulations [38]. Nevertheless, teachers’ power is restricted by external accountability introduced by neoliberal education policies, so they may be pressured to manage emotions via surface and deep acting rather than the expression of naturally felt emotions. External accountability is inclined to make schools and teachers more answerable to students and students’ parents because it usually involves the evaluation of their performance with standardized performance indicators and assessment, and the results are then provided to the public for school selection [39]. In this situation, schools tend to develop market-oriented management that values the satisfaction of students and students’ parents because satisfaction implies school quality, effectiveness, and competitiveness [34]. Thus, teachers are treated as service workers who have the responsibility to satisfy the students, and parents as clients who have the right to make requests and criticisms of them. In this teacher–student/parent relationship, therefore, teachers are pressured to manage their emotions, such as being polite and continuing to smile through surface and deep acting, to make the students and parents feel good [7].

Moreover, studies suggest that external accountability tends to bureaucratize schools because bureaucratization increases school effectiveness and efficiency in responding to external accountability demands [2]. Due to bureaucratization, there are stricter managerial mechanisms for monitoring teachers’ work in schools [40]. Thus, teachers may be required to display appropriate emotions via surface or deep acting to align with the expectations and requirements of managerial mechanisms, thereby gaining favorable evaluations [7,41]. For instance, they may choose to hide or suppress negative emotions and keep working without overt complaints, although they are dissatisfied with some practices of school management because they are expected to be obedient to school bureaucracy. In some schools, teachers’ emotional activities in teaching are also evaluated by school administrators through classroom observations and student feedback, so teachers need to perform surface or deep acting in the classroom according to the evaluation criteria [42]. Accordingly, external accountability may encourage teachers to perform surface acting and deep acting but discourage them from expressing naturally felt emotions.

Because emotional labor, including surface acting, deep acting, and the expression of naturally felt emotions, may affect teacher burnout and be affected by external accountability, it may play a mediating role between external accountability and teacher burnout. Thus, the following hypotheses are formulated:

**Hypothesis 3 (H3).**  *Surface acting plays a mediating role between external accountability and teacher burnout*.

**Hypothesis 4 (H4).**  *Deep acting plays a mediating role between external accountability and teacher burnout*.

**Hypothesis 5 (H5).**  *The expression of naturally felt emotions plays a mediating role between external accountability and teacher burnout*.

## 6. Chain Mediation of Internal Accountability and Emotional Labor

According to Hochschild [35], workers’ emotionality is disciplined by emotional rules prescribing how they should feel and display their emotions in the workplace. In education, for instance, teachers are subject to certain emotional rules, such as avoiding the expression of extreme emotions, hiding negative emotions but maintaining positive emotions, and being enthusiastic and passionate about students and subject matter [42,43]. Zembylas [42] illustrates that these emotional rules are incorporated into teacher professionalism. In other words, if teachers fail to feel or display the prescribed emotions appropriately, they will be perceived as unprofessional. As a result, they need to manage their emotions accordingly.

Therefore, emotional rules function as internal accountability that disciplines the emotionality of teachers. In other words, when emotional rules are present, teachers consciously or unconsciously experience internal accountability demands to perform surface acting and deep acting to meet emotional expectations and demands associated with teacher professionalism [28]. Thus, internal accountability may be positively related to emotional labor strategies. On the other hand, internal accountability may be negatively related to the expression of naturally felt emotions because emotional rules normatively require teachers to suppress their genuine emotions [41].

Since international accountability should be associated with the three emotional labor strategies and emotional labor strategies should be related to teacher burnout, there may be chain mediation effects on the relationship between external accountability and teacher burnout. Thus, the following hypotheses are formulated:

**Hypothesis 6 (H6).**  *Internal accountability and surface acting play a chain* *mediating role between external accountability and teacher burnout*.

**Hypothesis 7 (H7).**  *Internal accountability and deep acting play a chain mediating role between external accountability and teacher burnout*.

**Hypothesis 8 (H8).**  *Internal accountability and the expression of naturally felt emotions play a chain mediating role between external accountability and teacher burnout*.

## 7. Materials and Methods

### 7.1. Participants

When setting the statistical power to 0.8 and effect size = 0.5, with a 95% confidence interval and a two-tailed independent samples *t*-test the sample size should be above 600. Therefore, in this study, 814 primary and secondary teachers were sampled across 10 provinces (3 northern provinces, 3 mid-regional provinces, and 4 southern provinces) in China. Participating teachers were randomly selected from the database of the training center affiliated with a renowned normal university. Each teacher enrolled in the training center was given a unique identification number. These numbers were then fed into a computerized random number generator to produce a random sequence for selection. Selected participants received a text message with a link to an anonymous online survey questionnaire. To reduce social desirability bias, scale names were concealed. In total, 790 questionnaires were finally collected. After eliminating 55 invalid responses, 735 valid questionnaires (93.04% valid rate) were analyzed. Invalid questionnaires had either short answer times (under 240 s) or extreme scoring (all highest or lowest). Table 1 presents basic demographic information for the 735 participants.

### 7.2. Measures

#### 7.2.1. External and Internal Accountabilities

External and internal accountabilities were measured by the Chinese Version of the Personal Accountability Measure (PAM-Ch) [44]. This consists of 13 items measuring teachers’ perceptions of external accountability (5 items) and internal accountability (7 items). Sample items were “give yourself a report on the extent to which you reached your goals at work” (external) and “be responsible for using professional knowledge in your work” (internal). All items were rated on a 5-point Likert scale from 1 (completely disagree) to 5 (completely agree). Lower scores indicate lower levels of accountability.

#### 7.2.2. Teacher Emotional Labor

Teacher emotional labor was measured using the 13-item Teacher Emotional Labor Scale [33]. The TELSS contains three subscales assessing different emotional labor strategies: surface acting (6 items), deep acting (4 items), and expression of naturally felt emotions (3 items). Participants responded on a 5-point Likert scale from 1 (completely disagree) to 5 (completely agree). Sample items were “I put on a ‘show’ or ‘performance’ when interacting with students or their parents” (surface acting), “I try to actually experience the emotions that I must show to students or their parents” (deep acting), and “The emotions I show students or their parents match what I spontaneously feel” (expression of naturally felt emotions).

#### 7.2.3. Teacher Burnout

Teacher burnout was assessed with the 15-item Chinese version [45] of the Maslach Burnout Inventory-Educators Survey [13]. It was measured with a 7-point Likert scale from 1 (completely disagree) to 7 (completely agree). A sample item is “I can easily understand students’ feelings”. Lower scores indicate lower burnout.

#### 7.2.4. Control Variables

Gender, school phase, school quality, highest education, years of teaching (the number of years participants have been teaching, M = 13.42, SD = 10.97), and homeroom teacher status were controlled in this study because these demographic attributes may affect burnout and emotional labor [46]. For example, from the teacher life cycle perspective, burnout dropped significantly with increasing teaching age.

### 7.3. Data Analysis

First, the Harman single-factor test and variance inflation factor (VIF) values were examined to ensure that there was no serious common method deviation or multicollinearity concern. Then, the chain mediation effects were tested using bias-corrected bootstrapping with 5000 resamples to compute 95% confidence intervals (CIs). If the 95% CI for the indirect effect estimate excluded zero, the mediation effect was considered statistically significant. Multiple regression analyses were also conducted to identify the coefficients between major variables in the chain mediation model incorporating (1) control variables (gender, school phase, school quality, highest education, years of teaching, homeroom teacher status); (2) an independent variable (external accountability); (3) mediators (internal accountability, surface acting/deep acting/expression of naturally felt emotions); and (4) the outcome variable burnout. Hypotheses were tested along the line, and SPSS 26 and Process 3.4 were applied for data analysis.

## 8. Results

Prior to testing the serial mediation model, Harman’s single-factor test was conducted to assess common method variance. The results indicated eight factors with eigenvalues greater than 1, explaining 77.33% of the variance. The first factor explained only 33.73% of the variance, which is below the common threshold of 40%, which may indicate serious common method bias. Additionally, variance inflation factor (VIF) values for all predictor variables were under 5, suggesting no issues with multicollinearity among the variables.

### 8.1. Descriptions and Correlations

Table 2 shows the means, standard deviations, Cronbach’s alphas, and partial correlations between major variables, controlling for gender, school phase, school quality, highest education, years of teaching, and homeroom teacher status. The Cronbach’s alphas of all the key variables are above 0.80, indicating the high credibility of each measurement. Since the means of each dimension of accountability vary (3.93 for external accountability and 4.33 for internal accountability), the composited score may not fully represent each dimension. Therefore, it is worth examining closely and analyzing how external accountability and internal accountability function differently. Specifically, external and internal accountabilities are negatively and significantly related to burnout: preliminarily, H1 is not supported. In addition, external and internal accountabilities are both negatively and significantly related to surface acting, but positively and significantly related to deep acting and the expression of naturally felt emotions. Burnout is positively and significantly related to surface acting and deep acting, but negatively and significantly related to the expression of naturally felt emotions.

### 8.2. Chain Mediation Analysis

Three chain mediation models were tested to examine H2-H8, while the coefficients between major variables and their significance were calculated through multiple regressions but not tabulated: (1) external accountability—internal accountability—surface acting—burnout; (2) external accountability—internal accountability—deep acting—burnout; and (3) external accountability—internal accountability—expression of naturally felt emotions—burnout.

**Figure 1:**
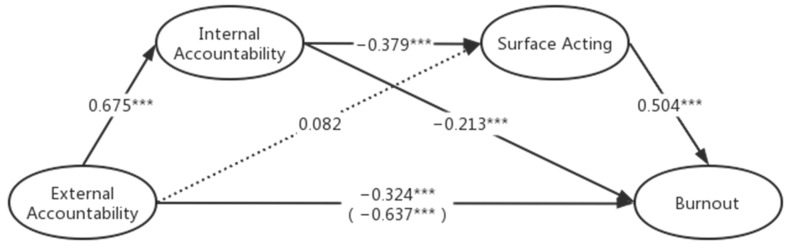
The relationship between external accountability and burnout mediated by internal accountability and surface acting. Note: * *p* < 0.05; ** *p* < 0.01; *** *p* < 0.001.

Table 3 shows the bootstrap results for the first chain model (the effect was considered statistically significant if the confidence interval excluded zero): external accountability—internal accountability—surface acting—burnout. The direct effect between external accountability and burnout is significantly negative (−0.324, [−0.446, −0.201]). H1 is supported. Path EA-IA-BO is significant (−0.143, [−0.227, −0.062]). H2 is supported. Path EA-SA-BO is not significant (−0.041, [−0.117, 0.029]). H3 is not supported. Path EA-IA-SA-BO is significant (−0.129, [−0.181, −0.080]). H6 is supported. The coefficients and significance are shown in Figure 1.

**Figure 2:**
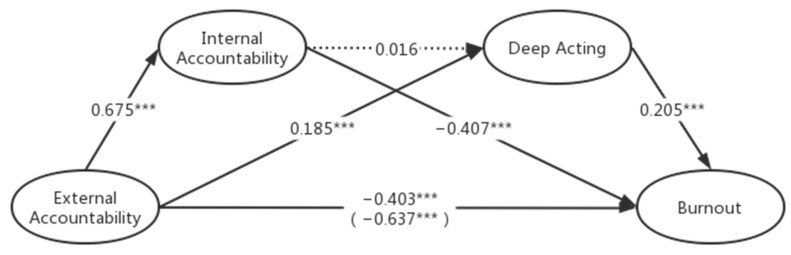
The relationship between external accountability and burnout mediated by internal accountability and deep acting. Note: * *p* < 0.05; ** *p* < 0.01; *** *p* < 0.001.

Table 4 shows the bootstrap results for the second chain model (the effect was considered statistically significant if the confidence interval excluded zero): external accountability—internal accountability—deep acting—burnout. The direct effect between external accountability and burnout is significantly negative (−0.403, [−0.541, −0.264]). H1 is supported. Path EA-IA-BO is significant (−0.274, [−0.367, −0.180]). H2 is supported. Path EA-DA-BO is significant (0.037, [0.009, 0.074]). H4 is supported. Path EA-IA-DA-BO is not significant (0.002, [−0.014, −0.020]). H7 is not supported. The coefficients and significance are shown in Figure 2.

**Figure 3:**
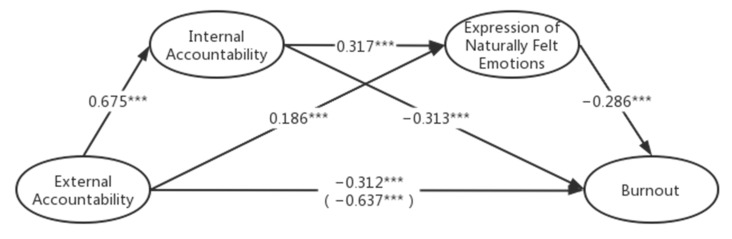
The relationship between external accountability and burnout mediated by internal accountability and the expression of naturally felt emotions. Note: * *p* < 0.05; ** *p* < 0.01; *** *p* < 0.001.

Table 5 shows the bootstrap results for the third chain model (the effect was considered statistically significant if the confidence interval excluded zero): external accountability—internal accountability—expression of naturally felt emotions—burnout. The direct effect between external accountability and burnout is significantly negative (−0.312, [−0.449, −0.175]). H1 is supported. Path EA-IA-BO is significant (−0.211, [−0.305, −0.121]). H2 is supported. Path EA-NFE-BO is significant (−0.053, [−0.100, −0.014]). H5 is supported. Path EA-IA-NFE-BO is not significant (−0.061, [−0.096, −0.032]). H8 is supported. The coefficients and significance are shown in Figure 3.

## 9. Discussion

According to the findings, this study shows that external and internal accountabilities are both negatively related to burnout. Moreover, it indicates that internal accountability, deep acting, and expression of naturally felt emotions can mediate the relationship between external accountability and burnout. Furthermore, the findings suggest that internal accountability and surface acting play a chain mediating role between external accountability and burnout. In addition, the chain mediating role of internal accountability and expression of naturally felt emotions between external accountability and teacher burnout is also identified by the study. Two major points require a deeper discussion, as follows.

### 9.1. Effects of External Accountabiliy

The labor process theory assumes that external accountability is an institutional force that alienates teachers from their labor, thereby making them feel negatively toward their work and prone to burnout [47]. To some extent, previous studies, especially qualitative studies (e.g., [24,48]), confirm the theoretical assumption because they show that external accountability in China altered teachers’ work routines and patterns, deskilled teachers, and intensified teachers’ work, leading to feelings of stress, exhaustion, and depersonalization among teachers, beginning in 2000. However, it is surprising that the assumption seems not to be supported by the present study due to the negative relationship between external accountability and teacher burnout.

The contradictory findings of the present study to those of previous studies may be attributed to the attention given to different measures of external accountability. Many previous studies have focused on external accountability measures such as educational inspections in China (e.g., [24]). This type of measure is enacted by the government to inspect whether schools and teachers implement educational law, regulations, and policies appropriately and the quality of their educational activities in China [10]. Accordingly, schools and teachers are managerially required to align their decisions, planning, actions, and outcomes with the state’s expectations and provide measurable evidence to support their claims of effectiveness, efficacy, and quality of education [48]. Therefore, these external accountability measures are inclined to deskill and intensify teachers’ work, thereby making them feel stressed, exhausted, depersonalized, and burned out. On the other hand, the PAM-Ch used to assess external accountability in the study tends to focus on the measure of standardized assessment, which is called performance-based accountability, holding teachers accountable for students’ performance on standardized tests and examinations. The focus is reflected by its items, such as “Be accountable for your students’ achievements”, “Be evaluated by whether your students improve their grades”, and “Obtain credit for the success of your classes”. To some extent, the literature implies that teachers in China tend to be committed to performance-based accountability and respond to it positively [9,21] for two possible reasons.

First, the education system in China rewards teachers with excellent performance in teaching, such as by giving them an honorable title to recognize their competence [49] and offering them more compensation or bonuses based on their merits [50]. Therefore, as Zhang and Tsang’s [51] study illustrates, teachers may feel joy toward performance-based accountability because they perceive that it provides opportunities for them to earn recognition and financial awards if they can produce high student scores on tests and examinations. Accordingly, teachers may be more resilient to the demands of performance-based accountability, and thereby better able to cope with burnout symptoms. Second, Chinese societies have a long history of examination culture that makes people prefer to evaluate teachers’ competence based on students’ performance on tests and examinations [21]. As a result, teachers in China commonly believe that their major responsibility is teaching to the test, in addition to molding the students into persons of good character [48]. In this sense, teachers are likely to take performance-based accountability for granted, be more committed to it, and hold a more positive attitude toward it, as influenced by the culture, thereby making them less at risk of burnout.

These two explanations for the positive relationship between external accountability and teacher burnout further imply two interpretations of the mediation effects of internal accountability on the relationship. First, as labor process theory suggests, the state attempts to discipline teachers’ subjectivity to obtain their consent and reduce their negative responses to stressful and alienated work conditions by promoting new professionalism based on neoliberal values of education, such as standardized tests and measurable outcomes [16,25]. In other words, states attempt to managerially push teachers to be internally accountable based on external accountability demands [9]. Alternatively, internal accountability can be about the traditional examination culture, which is not a normative mechanism initiated by the state. As mentioned, examination culture is deeply rooted in Chinese societies and has already had a significant influence on teachers’ subjectivity. Thus, teachers are likely to be culturally committed to teaching to the test. When the state exercises external accountability measures such as standardized assessments, the effects of the examination culture on teachers may be reinforced and reaffirmed [21]. In other words, the medication effects can suggest that teachers feel the responsibility to hold themselves accountable for the success of their students on tests and examinations because of the traditional culture instead of the new professionalism promoted by the state.

### 9.2. Roles of Emotional Labor

According to Brook [36] and Tsang [52], emotional labor, especially the strategies of surface acting and deep acting, is alienated labor. Therefore, surface acting and deep acting should be positively related to negative psychological outcomes because emotion management is forced and performed in line with structural demands of external and internal accountabilities; in contrast, as nonalienated labor enables expression of genuine emotions, expression of naturally felt emotions should be negatively related to negative psychological outcomes, but may be discouraged by external and internal accountabilities as they prescribe how teachers display emotions appropriately [7,41]. Nevertheless, the study does not fully support these claims of emotional labor theory. In contrast to the theoretical expectations, for example, the findings show that external and internal accountabilities are positively related to the expression of naturally felt emotion, external accountability does not directly affect surface acting, and internal accountability is negatively related to surface acting.

To explain the unexpected findings, we need to understand the Chinese emotional rules of teaching. In addition to those emotional rules commonly shared by teachers in many societies, such as hiding extreme emotions, maintaining positive emotions, and being passionate about teaching and students [42,43], Yin and Lee [53] find that the Chinese emotional rules of teaching also underline the instrumentalization of emotions. This emotional rule requires teachers to instrumentally use their emotions, whether genuine or fake, to ensure a smooth and effective teaching process. Therefore, in situations where teachers are held accountable for students’ success in tests and examinations by external or internal measures, the expression of naturally felt emotions may be favored if their genuine emotions can contribute to desirable teaching consequences (e.g., genuinely showing anger may help control classroom order to facilitate smooth classroom teaching and learning, and genuinely sharing joy with students may help maintain students’ motivation and commitment to learning). Accordingly, both external and internal accountabilities can be positively associated with the expression of naturally felt emotions.

On the other hand, if teachers perceive that their genuine emotions cannot help them achieve teaching goals, they may intentionally hide their genuine emotions and express fake ones [54]. According to the findings, teachers in China tend to prefer using deep acting rather than surface acting in response to the demands of external and internal accountabilities. A plausible explanation for the preference for deep acting is that teachers who employ deep acting to convey emotions are more likely to engender a belief in the authenticity and sincerity of their displayed emotions among students [55]. When teachers are perceived as authentic and sincere, students are more likely to be influenced by them, leading to increased motivation and engagement in learning processes [56]. Therefore, deep acting may be a more desirable strategy to manage emotions in teaching than surface acting [57]. Thus, compared with surface acting, the use of deep acting may be strengthened when teachers are required to teach to the test by external or internal accountability. This may be the reason why external accountability has no significant and direct effects on surface acting and why internal accountability is negatively related to surface acting.

## 10. Conclusions

In the neoliberal contexts of education, teachers are generally reported to be at risk of burnout because of intensive accountability demands [2,15]. However, the existing studies have tended to focus on the external form but ignore the internal form of accountability affecting teacher burnout. Moreover, although emotional labor is recognized as a significant factor predicting teacher burnout [7], there is little research investigating its role in the relationship between external and internal accountabilities and teacher burnout. In contrast to previous studies, the present study investigated how teacher burnout is affected by external and internal accountabilities and emotional labor strategies of surface acting, deep acting, and expression of naturally felt emotions in China from labor process theory and emotional labor theory. According to the findings, the relationships between these constructs are more complicated than expected.

To some extent, the findings of the study prompt us to revisit the basic assumptions of labor process theory and emotional labor theory. First, the theories assume that external and internal accountabilities are coercive mechanisms because they are used to explicitly or implicitly disempower teachers from exercising control over their labor, including emotional labor, through managerial or normative mechanisms, thereby alienating them from their labor and resulting in negative psychological outcomes such as burnout [15,16,25]. Nevertheless, the findings suggest that accountabilities can also be enabling mechanisms, as they may increase teachers’ labor power, at least the labor power to express naturally felt emotions, leading to a low level of burnout.

Second, they assume that emotional labor, especially surface acting and deep acting, is alienated labor, not only because it can affect workers’ psychological well-being but also because it is emotion management forced by accountability measures in the neoliberal era [36]. However, the study does not fully confirm this assumption since the findings show that surface acting is negatively associated with both external and internal accountabilities. In other words, teachers may perform surface acting not because of the structural demands of accountability mechanisms but for other reasons, such as the lack of sophisticated skills to authentically express those prescribed emotions in teaching [57]. Accordingly, surface acting may not be forced labor in teaching.

Third, it is assumed that the expression of naturally felt emotions is a spontaneous expression of genuine emotions [37]. However, the findings imply that the expression of naturally felt emotions may not be as spontaneous as expected among teachers in China because it may be positively affected by external and internal accountabilities. In other words, teachers may instrumentally use this strategy to respond to the demands of external and internal accountabilities [53]. Accordingly, it can be regarded as managerially or normatively forced emotional emotion management.

Finally, although it is commonly assumed that external and internal accountability are two distinct forms [4,26,44], the findings raise a question regarding the essentiality of differentiating between them while examining the mechanisms underlying teacher burnout, because of the moderate correlation observed between external and internal accountability and the similar patterns of their effects on teacher burnout indicated by the study. The findings suggest that external and internal accountabilities may operate on a continuum and function more as a combined factor rather than two separate entities. In other words, teachers may experience a blend of both forms of accountabilities, contributing to their experiences of burnout. If this is the case, it suggests the existence of a complex and dynamic interplay between external and internal accountabilities, where external accountability can serve as either a predictor or a dependent variable of internal accountability, and vice versa.

Accordingly, further development of labor process theory and emotional labor theory is required to advance our understanding of the complicated relationships between external and internal accountabilities, emotional labor, and teacher burnout in China. Based on the above discussions, it is noted that the reason why the theories cannot entirely explain the findings of the study is the neglect of the influences of Chinese culture, such as examination culture. Therefore, scholars should pay greater attention to societal culture and investigate how societal culture works with neoliberalism, affecting accountabilities, emotional labor, teacher burnout, and their relationships in China. Moreover, as the discussion notes, focusing on different types of external accountability measures can lead to different conclusions regarding the relationships between accountability, emotional labor, and teacher burnout. Therefore, further studies should differentiate the effects of different types of external accountability measures while examining the impacts of external accountability on emotional labor and teacher burnout.

The findings of this study have significant implications for education policymakers and school leaders who are responsible for creating supportive environments for teachers’ work and well-being. For example, they should enact policies or administrative practices to strike a balance between external and internal pressures and demands of accountability and promote international accountability based on intrinsic motivation and a sense of autonomy. These can be achieved through the development of policies that value teacher autonomy, encourage professional growth, and provide supportive structures to help teachers navigate external accountability pressures and demands. Moreover, as emotional labor can mediate the relationship between accountabilities and burnout, improving teachers’ emotional labor skills is important. Thus, policymakers and school leaders should prioritize the inclusion of emotion management strategies in teacher professional development programs and trainings. Furthermore, the chain mediation models identified in the study suggest that interventions targeting specific points in the accountability–emotional labor–burnout chain can have cascading effects on overall teacher well-being. Therefore, they should consider enacting comprehensive well-being interventions, such as providing resources for teachers to develop their internal accountability, fostering supportive relationships among colleagues, and designing institutions to encourage the performance of genuine emotional expression rather than surface acting in teaching.

Several limitations of this study are acknowledged. First, due to its cross-sectional design, the study can only identify correlations and cannot establish a causal relationship between the variables. Thus, further studies should employ a longitudinal design to examine the causal relationships. Second, external and internal accountabilities are institutional-level constructs, while emotional labor and burnout are individual-level constructs, so the absence of a multilevel analysis may limit the validity of the research findings. Thus, further studies should analyze the relationships between the constructs via advanced statistics such as multilevel structural equation modeling. To achieve this, researchers should randomly select participants using a stratified multistage sampling strategy. Finally, this study was conducted in China, where the sociocultural contexts are different from those in Western countries. Therefore, caution should be taken when generalizing the results to other contexts. Future studies should be conducted in different sociocultural contexts to increase the generalizability of the findings.

## Figures and Tables

**Table 1:** Basic information of the participants (*n* = 735).

		Number	Rate
Gender	Female	541	73.6%
	Male	194	26.4%
School Phase	Primary School	562	76.5%
	Middle School	74	10.1%
	High School	99	13.5%
School Quality	Key School	295	40.1%
	Ordinary School	440	59.9%
Highest Education	Associate Degree	51	6.9%
	Undergraduate	621	84.5%
	Graduate	63	8.6%
Homeroom Teacher Status	Yes	328	44.6%
	No	407	55.4%

**Table 2:** Means, standard deviations, Cronbach’s alphas, and correlations (*n* = 735).

	M	SD	α	EA	IA	SA	DA	NFE	BO
EA	3.93	0.74	0.90	-					
IA	4.33	0.70	0.97	0.715 ***	-				
SA	2.14	1.00	0.94	−0.270 ***	−0.328 ***	-			
DA	3.13	0.87	0.83	0.144 ***	0.104 ***	0.411 ***	-		
NFE	3.68	0.86	0.80	0.341 ***	0.366 ***	−0.262 ***	0.237 ***	-	
BO	2.01	1.12	0.87	−0.435 ***	−0.439 ***	0.561 ***	0.100 ***	−0.373 ***	-

Note: EA external accountability, IA internal accountability, SA surface acting, DA deep acting, NFE expression of naturally felt emotions, BO burnout. * *p* < 0.05; ** *p* < 0.01; *** *p* < 0.001.

**Table 3:** Bootstrap results for EA-IA-SA-BO.

Model Summary					
R	R-sq	MSE	F	*p*	
0.667	0.443	0.71	64.26	0.000	
Direct Effect—Bootstrap					
Effect	SE	t	*p*	LLCI	ULCI
−0.323	0.06	−5.19	0.000	−0.446	−0.201
Indirect Effect—Bootstrap					
	Effect	SE	LLCI	ULCI	
Total	−0.314	0.049	−0.412	−0.218	
EA-IA-BO	−0.143	0.042	−0.227	−0.062	
EA-SA-BO	−0.041	0.037	−0.117	0.029	
EA-IA-SA-BO	−0.129	0.025	−0.181	−0.080	

Note: EA external accountability, IA internal accountability, SA surface acting, DA deep acting, NFE expression of naturally felt emotions, BO burnout, LLCI lower level confidence interval, ULCI upper level confidence interval, SE standard error (same for the following tables).

**Table 4:** Bootstrap results for EA-IA-DA-BO.

Model Summary					
R	R-sq	MSE	F	*p*	
0.542	0.294	0.901	64.262	0.000	
Direct Effect—Bootstrap					
Effect	se	t	*p*	LLCI	ULCI
−0.403	0.070	−5.709	0.000	−0.541	−0.264
Indirect Effect—Bootstrap					
	Effect	BootSE	BootLLCI	BootULCI	
Total	−0.234	0.049	−0.332	−0.140	
EA-IA-BO	−0.274	0.047	−0.367	−0.180	
EA-DA-BO	0.037	0.016	0.009	0.074	
EA-IA-DA-BO	0.002	0.008	−0.014	0.020	

**Table 5:** Bootstrap results for EA-IA-NFE-BO.

Model Summary					
R	R-sq	MSE	F	*p*	
0.557	0.310	0.880	36.347	0.000	
Direct Effect—Bootstrap					
Effect	se	t	*p*	LLCI	ULCI
−0.312	0.069	−4.471	0.000	−0.449	−0.175
Indirect Effect—Bootstrap					
	Effect	BootSE	BootLLCI	BootULCI	
TOTAL	−0.325	0.048	−0.424	−0.232	
EA-IA-BO	−0.211	0.047	−0.305	−0.121	
EA-NFE-BO	−0.053	0.022	−0.100	−0.014	
EA-IA-NFE-BO	−0.061	0.016	−0.096	−0.032	

## Data Availability

The data presented in this study are available upon request from the corresponding author. The data are not publicly available due to confidentiality and research ethics.
